# Emerging zoonotic diseases and COVID-19 pandemic: global Perspective and Indian Scenario

**DOI:** 10.1097/MS9.0000000000001057

**Published:** 2023-07-07

**Authors:** Mainak Bardhan, Ishita Ray, Shubhajeet Roy, Parjanya Bhatt, Suyog Patel, Sucharu Asri, Sanobar Shariff, Anagha Shree, Saloni Mitra, Priyanka Roy, Ayush Anand

**Affiliations:** a Indian Council of Medical Research, New Delhi; b Miami Cancer Institute, Baptist Health, South Florida, USA; c Mahatma Gandhi Memorial Medical College, Indore; d King George’s Medical University, Lucknow; e B.J. Medical College, Ahmedabad; f SGT Medical College Hospital and Research Institute, Haryana; g Yerevan State Medical University, Armenia; h OO Bogomolets National Medical University, Kyiv, Ukraine; i Department of Labor, Government of West Bengal, Kolkatta, West Bengal, India; j B. P. Koirala Institute of Health Sciences, Dharan, Nepal

**Keywords:** COVID-19, langya Virus, monkeypox, nipah virus, zoonotic diseases

## Abstract

The current coronavirus disease 2019 (COVID-19) pandemic is one example of the scores of zoonotic diseases responsible for various outbreaks resulting in the deaths of millions of people for centuries. The COVID-19 pandemic has broken the age-old healthcare infrastructure and led to utter chaos. In the shadow of this pandemic, other zoonotic infections like the nipah virus, monkeypox, and langya virus, to name a few, have been neglected. Hence, outbreaks caused by such zoonotic viruses are rising in their endemic areas, like the Indian subcontinent. The mortality and morbidity due to such zoonoses are greater than usual due to the shortage of healthcare professionals caused by the COVID-19 crisis. Due to the lack of vaccines and therapeutics directed against this viral infection, treatment of patients is limited to supportive management and prevention, making preparedness for these potential zoonotic viral outbreaks essential. This paper highlights some of these zoonotic infections, which perpetuated and wreaked havoc while the world was occupied with containing the COVID-19 pandemic.

## Introduction

Highlights

- Unchecked emerging zoonotic disease during coronavirus disease can lead to the next pandemic.
- Lack of infrastructure, healthcare workforce, awareness, and preventive strategies contribute to the increased mortality and morbidity associated with the zoonotic disease.
- An active role of local and international stakeholders is required to prevent zoonotic disease transmission.

According to the WHO, zoonotic diseases are infections transmitted from nonhuman species to humans and can be bacterial, fungal, viral, or parasitic^1^. Some of the common examples of zoonotic diseases are bacterial (salmonellosis, brucellosis, plaque, and leptospirosis), fungal (cryptococcus and histoplasmosis), parasitic (leishmaniasis, hydatid disease, schistosomiasis, and toxoplasmosis), and viral (rabies, influenza, and yellow fever), etc. Animals have played a paramount role in the development of human civilization in many aspects of living, like food, shelter, transport, and business. This has led to increased contact between humans and animals, facilitating the transmission of these diseases^2–7^.

History is the witness of many zoonotic pandemics, such as the Spanish flu pandemic (1918), the Black Death pandemic due to the bubonic plague (1346–1353), the Plague of Justinian (541–543), and many more^8^. A more recent zoonotic virus is the zoonotic virus named nipah virus (NiV) emerged in 1999 in Malaysia^9^. It is known to cause deadly encephalitis in children and is transmitted through raw date palm saps through bats^10^. The 2019–2020 severe acute respiratory syndrome coronavirus 2 (Sars-CoV-2) pandemic caused by coronavirus is another example of how a zoonotic virus speculated to emerge from a live animal market in the Wuhan province in China caused an unforeseen pandemic that affected the entire world.

India is a predominantly agricultural country, and animal husbandry is crucial to the economy. Many risk factors have created a surge in zoonotic diseases like population explosion, industrialization, deforestation, and urbanization^4–7,11^. This disturbance in the ecological system promotes animal contact with humans. Also, since India is a developing and resource-poor country, insufficient sanitation facilities, poor nutrition, and a lack of health education predispose the population to zoonotic diseases^12^. The COVID-19 pandemic was an ultimate blow to the Indian healthcare system, where other zoonotic diseases consumed the resources perpetuated relentlessly in its shadow. However, in some cases, the strict quarantine measures during the COVID-19 pandemic helped prevent the spread of other zoonotic diseases. Our article attempts to offer information about emerging zoonotic infections in India and the COVID-19 pandemic.

### Zoonotic diseases and zoonosis

**Figure 1:**
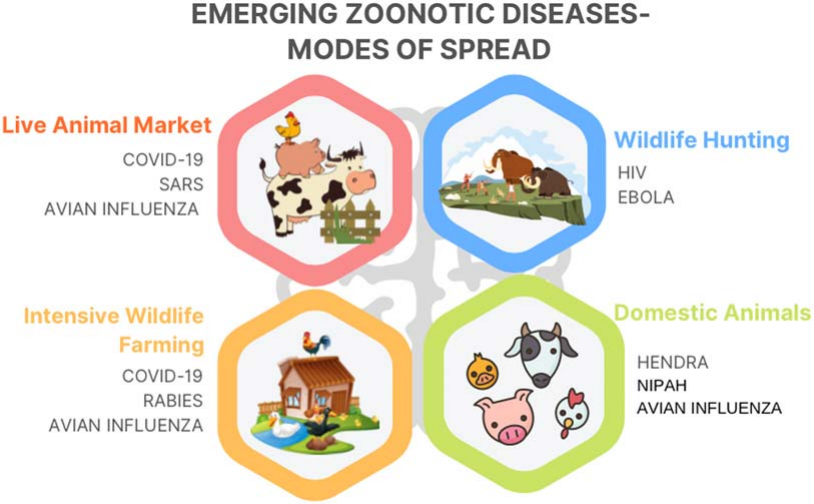
Modes of the spread of emerging zoonotic diseases. COVID-19, coronavirus disease 2019; SARS, severe acute respiratory syndrome.

According to the WHO, zoonosis is an infectious disease that spills from nonhuman species to humans. Zoonotic pathogens (Table 1) can fall under bacteria, viruses, parasites, or unconventional novel agents. They can spread to humans (Fig. 1) via direct contact or the oral route of food, water, or a vector animal^1^. The spillover of infectious agents from other species to humans is very unpredictable, making predicting pandemics caused by such zoonosis an arduous task. Morse *et al*.^13^ have discussed the Daszak model to understand zoonosis and the pandemic potential of pathogens. This model defines three stages of spillover —no human infection (stage 1; spillover), localized infection in humans (stage 2; spillover), and widespread transmission and global spread (stage 3). It is safe to assume that the frequency of stage 1 and stage 2 is high due to the large-scale exposure of humans to zoonotic pathogens in nature^14^.

**Table 1:** Classification of zoonotic pathogens based on pathogen type into bacterial, viral, fungal, and parasitic, along with their hosts

Bacterial pathogens-hosts	Viral pathogens-hosts	Fungal pathogens-hosts	Parasitic pathogens
Brucells- Racoon, Hare	Rabies- Dogs, bats	Cryptococcus and Histoplasmosis- Bats	Leishmaniasis- Sandfly
Leptospira- Rats, Cattle, Buffalo	Influenza- Bats, Camels		Hydatid Disease- Dogs
Salmonella- Poultry and Eggs	Dengue and Chikunguniya- Aedes Mosquito		Schistosoma- Snails
Bubonic Plague- Rats	Japenese Encephalitis Virus- Pond Herons, Cattle Egrets		Toxoplasma- Cats
	Hanta Virus- Bandicots, Rats, Shrews		
	Crimean Congo Hemorrhagic virus- Shrews, bats		
	Kyasanur Forest Disease Virus- Sick/Dying Monkeys, Birds, Ticks		

Parrish *et al*. defined three phases of the emergence of viral diseases through zoonotic spill over, spillovers into ‘dead-end’ hosts, the first isolated infection of a host organism with no chance of transmission, and spillovers that result in local chains of transmission in a small host population group endemically before epidemics, and epidemic or sustained endemic disease^15^. Many variables affect the successful disease emergence through zoonosis. These are the type of zoonotic species, the basal immunity of the host species, the degree of contact between the two species, the host barrier to infections, viral factors that potentiate the spread of infection like virulence, and the level of mutagenicity of the virus species. It is seen that infections in animal species more closely related to the Homo sapiens, like primates, tend to spill over faster to the human population and should be considered while determining the pandemic potential of pathogens. Live meat markets, high levels of deforestation, and meat consumption from exotic species like bats, monkeys, etc. also increase the risk of zoonotic spillover^16^.

Bats harbor more than 60 species of viruses like rabies virus and other Lyssavirus (Family Rhabdoviridae), Hendra and NiV (Paramyxoviridae), Marburg and Ebola viruses (Filoviridae), MERS-CoV and SARS-CoV (Coronaviridae) to name a few^17^. A complex association between the host, the agent, and the environment has provided many ways to transmit disease organisms to humans. These human spillovers can lead to small outbreaks, halting the disease process if controlled and studied well. If not, this may become a concern for affecting a large world population in pandemics; COVID-19 is a recent example. Like bats, several animals nurture zoonotic organisms’ life cycles, which can become a deadly problem in the future. Being an agricultural hub, India can become a study field to have more significant insights into understanding the chains and cycles of zoonoses.

### Nipah virus

NiV is a Pteropus fruit bat-borne pathogen and a genus Henipavirus (family Paramyxoviridae) member. It causes infections with high mortality in humans, like encephalitis, and is one of the world’s rising deadliest zoonotic viruses. NiV cases have been reported in various countries across South Asia, like Singapore, Malaysia, Bangladesh, and India^10^. The virus was called Nipah after the Malaysian hamlet of Sungai Nipah, where the first outbreak occurred in 1998^9^. The virus spreads to humans via pigs, the virus’s intermediary hosts. Bangladesh, Singapore, and Malaysia reported recurrent NiV outbreaks when India encountered its first outbreak in 2001 in Siliguri, West Bengal, then subsequent cases in the Naidu district of West Bengal in 2007. In Kerala’s Kozhikode district, there was another fatal NiV upsurge in 2018. The index patient acquired the NiV virus from fruit-eating bats, and the fatality rates went through the roof in all three outbreaks^10^.

In recent history, a NiV outbreak was seen in Kozhikode, Kerala, in September 2021, where a 12-year-old boy presenting with acute encephalitis tested positive for NiV and died from the infection. Reaching the diagnosis of NiV infection is difficult as there are many overlaps between the presentations of other acute respiratory infections and encephalitis syndromes. The SARS-CoV-2 pandemic made diagnosing zoonotic diseases even more challenging due to the overlapping clinical features between the infections. A promptly deployed outbreak containment response and the strict biosafety practices followed during the COVID-19 pandemic in Kerala possibly helped to prevent further transmission of NiV and to restrict the outbreak^18^.

Transmission through respiratory droplets of an infected person and contaminated date palm sap was also seen in India; hence, control measures to ensure the safety of date palms made available for human consumption would help curb future incidents of NiV infection. Anthropogenic activities and other agricultural activities such as intensive livestock farming, clearance of land for farming and grazing, human interference with natural habitats, and the coexistence of farm animals with people are a few reasons for zoonotic outbreaks^19^. Genetic factors also play a prominent part in the onset of NiV outbreaks. Researchers using genome sequencing have found two strains of NiV, NiVB, and NiVM. They are in charge of spreading diseases around the world. Bangladesh and India cited NiVB as the virus’s origin, while Malaysia reported NiVM as the leading source^10,19^. NiV fulfills the criteria of being a potential bioterrorism agent due to its high virulence, animal-to-animal and human-to-human spread, and the significant economic loss and morbidity that the virus causes. It is categorized as a category C agent on a list of bioterrorism agents by the Centers for Disease Control and Prevention^20^. Hence, it is essential to prevent future outbreaks by controlling the encroachment of humans into the animal territory, preventing fruit-eating bats from gaining access to products meant for human consumption, and using safety practices during pig rearing, which act as intermediate hosts of the virus and vector and sentinel surveillance for early detection of new cases of NiV infection.

### COVID-19

Coronaviruses, first identified in the 1960s, are known to infect Mammals and Aves. A study on 425 COVID-19 patients from Wuhan showed that 55% had a linkage with Huanan Seafood Wholesale Market^21^. SARS-CoV-2 was said to have its origins in bats, with palm civet cats being the intermediate host^22^. But, civet cats (intermediate host during SARS-CoV) have ACE2R recognizing only SARS-CoV and not SARS-CoV-2, thus making it a distant possibility^23^. Molecular analysis showed that SARS-CoV-2 had similarities with the horseshoe bat coronavirus from Yunnan^24^. But, for bat viruses to spill over and infect humans, the dependence is on the Spike glycoprotein’s receptor binding domain^25^. RaTG13 genome of bat coronavirus shares 1/5 important amino-acids present in the receptor binding domain, but pangolin CoV shares all 5/5^26^. Also, the skin scales of pangolins sold in the Huanan Seafood Market find use in Chinese medicine^27^. Genomes of Malyan pangolins, six from Guangxi: GX/P1E, GX/P3B, GX/P2V, GX/P4L, GX/P5E, and GX/P5L, and two from Guangdong: GD/P1L and GD/P2S had 85.5–92.4% sequence homology with SARS-CoV-2^28^. Pangolins are known to harbor Beta coronaviruses according to metagenomic profiling of CoVs^29^. This proves that the COVID-19 pandemic is a newly emergent zoonotic infection.

### Langya virus

The Paramyxoviridae family includes the Langya henipavirus (LayV). According to a recent study, although seropositivity was also found in goats and dogs, shrews may be the main natural reservoir for LayV^30^. Acute LayV was recently shown to be the sole pathogen infecting 35 patients in the Chinese provinces of Shandong and Henan between 2018 and 2021, with the latter part of the study overlapping with the COVID-19 pandemic; in 26 of these patients, no other pathogens were found. The clinical characteristics found in 26 patients included, but were not limited to, fever, lethargy, cough, anorexia, myalgia, nausea, headache, vomiting, thrombocytopenia, leukopenia, and decreased liver and kidney function. The transmission may be irregular due to the lack of interaction and shared history. Even if the sample group was insufficient to support claims of being transmitted by humans and zoonoses globally, it is essential to remember how the coronavirus spread throughout all continents. Good surveillance and awareness are required to prevent future hazards and pandemics.

### Monkeypox

One of the most virulent pox viruses, monkeypox, is typically found in tropical West and central African rainforests^31^. The Democratic Republic of Congo reported the first case of monkeypox in a human being in 1970. There are two clades in the virus’s phylogeny. One clade appeared in the Congo Basin of Central Africa, the other in West Africa. According to the WHO, there were a few confirmed cases of a monkeypox outbreak in the US in 2003, and the largest outbreak occurred in Nigeria in 2017. The organization also reported the virus’s exporting from African regions to nations like Israel, the UK, and Singapore. Monkeypox was first discovered in the United Kingdom on 7 May 2022, raising fears that the virus may spread and cause an outbreak, particularly amid the continuing COVID-19 pandemic. After that, the cases multiplied; by 10 June 2022, over 1500 cases from 43 nations, including those in Europe and North America, were reported. On 14 July 2022, India reported the first case in South Asia. There have been 10 confirmed cases thus far, including one locally transmitted case with no previous travel. The monkeypox virus can be transmitted by contact through contaminated skin, body fluids, or respiratory droplets. The virus spreads through intradermal injection, oral, and nasopharyngeal fluid exchange, and then swiftly multiplies at the inoculation site before moving on to adjacent lymph nodes. Fever, shivers, rash, headaches, muscle aches, aching joints, and weariness are some of the clinical features of the monkeypox illness^32^. Incidences of pneumonia, encephalopathy, sight-threatening keratitis, and recurring bacterial infections can result from monkeypox^33^. Given the timing of sexual contact, enhanced inguinal lymphadenopathy, and relapse of the rash, it is possible to have genital storage of the monkeypox virus, as has been shown with many other developing viruses. It is imperative to conduct more research in this area because it has immediate implications for using medical resources, improved patient and discharge, and transmission prevention. The pathogenicity of individuals with positive respiratory system swabs and crusty skin lesions remains unknown^34^. In the past, occasional incidents have been mentioned in reports. Several incidents have been connected to overseas travel or the importation of animals from Africa. The diagnosis was challenging since the virus was isolated to a specific region of the world when the initial cases were found in the other areas. This highlights the significance of identifying possible threats from zoonoses to humans. According to one idea, the smallpox virus left an ecological niche open for the monkeypox virus to occupy^35^. In the US, a smallpox treatment drug has also been approved to treat monkeypox. The immunizations employed during the smallpox eradication effort also provided immunity to monkeypox^33^. Avoiding infection using the smallpox vaccination JYNNEOS (Imvamune or Imvanex) is also possible^32^. Many people who have the condition experience mild courses that are self-limiting. Antivirals like tecovirimat and brincidofovir were among the available treatments. The smallpox vaccination used in the smallpox eradication campaign is akin to one vaccine made by Emergent BioSolutions. In contrast, the other, made by Bavarian Nordic, uses a nonreplicating variant of the vaccinia virus specifically created to have fewer adverse effects^36^.

To explain this return after 40 years of no known occurrences, Nguyen and colleagues proposed two primary mechanisms: Firstly, population mobility, violent conflicts, and deforestation have increased residents’ exposure to and interactions with forest animals. Secondly, since the 1970s, universal smallpox vaccination campaigns were terminated, and herd immunity has decreased. The two views, which are not mutually incompatible, represent the loss of two distinct spillover barriers^37^. One can wonder if the ongoing epidemic, urbanization, travel, and waning immunity encourage these microorganisms to occupy the space left by a virus that has been destroyed. Or is this just the beginning of a bonfire that could endanger people in the future? Whether this occurs spontaneously, due to human error, or for experimental purposes, there is a threat to civilization due to the lessons learned during the epidemic. To avert such accidents, there is a need for education and vigilant oversight by committed and dependable authorities.

### Other recent zoonotic diseases

Zoonotic diseases [Table 1], a constant threat to the world, are still underrated in many aspects. COVID-19 emerged as a hazard, leaving a bruise on the planet and an example to humankind about the potential threat zoonotic diseases could cause. Anthrax, Brucellosis, Bovine Tuberculosis, Cysticercosis, Rabies, Japanese Encephalitis (JE), Echinococcosis, Leptospirosis, Scrub Typhus, Toxoplasmosis, Scrub Typhus, and Nontyphoidal Salmonellosis are the most significant neglected zoonotic diseases, particularly in India. Apart from Nipah, major zoonotic diseases prevalent in India include Rabies, JE, Plague, Brucellosis, Toxoplasmosis, Leptospirosis, Kyasanur forest disease (KFD), Cysticercosis, Crimean Congo hemorrhagic fever, Echinococcosis^2^.

Scrub typhus, one of the vector-borne zoonotic diseases, originates from the bacteria Orientia Tsutsugamushi. This specific ailment plays a clinically significant role in Rickettsial infections worldwide. It is estimated that almost one million cases are seen globally in a year, with a high rise in the mortality rate. Scrub typhus exhibits symptoms like fever, cough, nausea, headache, breathlessness, and sometimes altered sensorium. Research has proved that scrub typhus can be interpreted in cases of undifferentiated febrile illness up to 35–50%, which might require hospitalization. One-third of the cases related to scrub typhus end up with multiorgan severe dysfunction, namely cardiac, renal, pulmonary, and hepatic complications.

Similarly, zika virus, another zoonotic disease, is due to a virus imparted by *Aedes aegypti* mosquitoes, which bite during the daytime. This virus was primarily detected in monkeys in the Zika forest in Uganda and gradually appeared in humans with time. The zika virus outbreak was noticed on the island of Yap in 2007 in the Pacific. It received the international community’s attention during its epidemic in Brazil in March 2015. Symptoms of this virus include headache, malaise, muscle, and joint pain, fever, rash, and conjunctivitis. Other complications are caused during pregnancy. Infants born with congenital zika syndrome have microcephaly and other congenital abnormalities. The zika virus may cause a pregnant woman to be prone to preterm birth and miscarriage. Guillain–Barre syndrome, neuropathy, and myelitis are a few of the neurological symptoms most commonly seen with the zika virus.

Dengue, the most popular arbovirus illness in humans, transmitted by female *Aedes* mosquito, is broadcasted in the headlines with a high incidence in India, especially from July to November. The upsurge of dengue fever is a potential threat to society, and a vital, timely treatment must be taken to prevent mortality.

Hydrophobia, another name for rabies, is a severe and deadly viral illness that affects the nervous system. Type 1 Lyssavirus is the culprit. Severe bites or scratches from rabid animals can cause harm to humans since the virus is widely present in their saliva. It is the only fatal communicable disease of man. The patient experiences intolerance to light, noise, and water due to the excitation of the sensory, motor, sympathetic, and mental systems. Postexposure prophylaxis includes local treatment of wounds, immunization, and antibiotics treatment^38^. Group B Flavivirus is the culprit behind the mosquito-borne illness known as JE. Glycoproteins of virus envelopes cross-react with neutralizing epitopes. The virus multiplies in local lymph nodes and invades the central nervous system through the blood. Prodromal symptoms are followed by acute encephalitis and late-stage sequelae^39^.

The febrile illness known as KFD is accompanied by hemorrhages. It is brought on by a flavivirus spread through a tick bite. Patients experience symptoms ranging from fever and headaches to myalgia, hemorrhages, and neurological symptoms^40^.

### The present scenario of zoonotic diseases during COVID-19

On 11 March 2020, officially marked the birth of the COVID-19 pandemic. Of the seven coronaviruses known to infect humans till now, two of them, that is, SARS-COV and MERS-COV, are zoonotic and have led to high-risk outbreaks. SARS-COV and MERS-COV have bats as primary hosts, palm civet cats, and dromedary camels as intermediate hosts^41^. Zoonotic diseases and other emerging infectious diseases usually occur due to the effectual interactions among populations of wildlife, livestock, and people within rapidly changing environments^42^. During the COVID-19 pandemic, due to stringent nonpharmaceutical measures, strict containment, and other suppression strategies incorporated by countries across the globe, there have not been any significant data indicating any notable increase or decrease in other zoonotic diseases. However, according to a recent study, there might have been a substantial increase in the overall cases of zoonotic diseases like anthrax, brucellosis, leptospirosis, and hydatid disease from 2021 in comparison to 2020 in China, perhaps due to the relaxation of nonpharmacological interventions in 2021^43^. The United States has also been prey to a few zoonotic outbreaks between 2020 and 2021. As of 29 September 2021, Salmonella outbreaks linked to small turtles were reported in 20 states and the District of Columbia. Eighty-seven illnesses, 32 hospitalizations, and one death was reported for the same^44^. Similarly, in November 2020, 18 people were infected with the Salmonella Muenster strain in eleven different states nationwide; 11 sick persons were hospitalized, but no fatalities were reported. It was discovered that the likely outbreak source was bearded pet dragons^45^. Monkeypox virus cases were also multiplied across 43 nations in June 2022. Not only this, KFD, also popularly known as monkey fever, poses a significant threat to the Kargal village in South India, especially after a large outbreak wreaked havoc in the neighboring village of Arlagodu in 2019, leaving three critically ill and two dead^46^. The above examples suggest that due to the high mutation rate and increased susceptibility to adaptation to new surroundings of the viruses, there is a high probability of the rise of new and old zoonotic diseases such as NiV, Leptospirosis, monkeypox virus, dengue, etc. during the COVID-19 pandemic^47^. Global health organizations and various nations have implemented health approaches like vaccination drives, surveillance, early warning systems, improved veterinary and animal health approaches, wildlife conservation and habitat protection, and one health (OH) approach. OH approach is the collaboration between local, national, and global experts from healthcare, public health, forestry, veterinary, and environmental to bring maximum health outcomes for humans, animals, and the environment by recognizing the interconnection between people, animals, plants, and their shared environment^48^. Despite these strategies, the monitoring and surveillance of various zoonotic diseases were disturbed during the pandemic, thus making it a pressing concern. In Developing countries like India, the OH approach is still in its embryonic stages^48^. Therefore, it is imperative that policies and regulatory measures be made to develop this approach further to avoid decreased monitoring, prevention, and surveillance of zoonotic disease occurrence in future pandemics. While there is no noteworthy report stating the COVID-19 pandemic to be the absolute cause of these zoonotic outbreaks in these countries, it leaves us to question the scenario of other zoonotic diseases during the pandemic and its major reasons. Adequate studies need to be conducted to analyze whether the COVID-19 pandemic had any potential contribution to the aggravation or decline of other zoonotic diseases.

### Challenges of zoonotic diseases amidst the COVID-19 pandemic

The COVID-19 pandemic is one of the many zoonotic-origin diseases, along with other emerging human infections whose origins lie in an animal host population. More research is needed to understand pathogenic diversity, a host-pathogen network of association, and host shifting, which can cause unprecedented human infections. We still have a limited understanding of the human-wildlife interface and the dynamics, which govern the spread of infectious pathogens from wildlife to humans resulting in new emerging diseases^49^.

Zoonotic diseases, amidst the pandemic, brought tremendous loss, be it loss of life, economy, or mere social chaos due to the preoccupation of the healthcare system’s efforts to deal with the COVID-19 crisis. Amongst them, late identification of zoonoses in humans, the absence of cooperation between medical doctors and veterinarians, a lack of knowledge about the disease, and a lack of awareness among the general population could be a real challenge and hinder preprepared efforts for known zoonotic infections. Inadequate measures in controlling international travel of infected cases across countries, a paucity of cooperation from the general public in following quarantine measures specified for the said zoonotic infection, and a dearth of strictness in implementing norms made by the healthcare administration sector have further boosted the spread of various zoonotic infections despite the ongoing COVID-19 pandemic^50^. Controlling the spread of diseases is very costly for developing or underdeveloped countries as there is a lack of funds or proper resources. For the past few decades, including during the pandemic, ‘new’ zoonosis has emerged. Lack of effective surveillance mechanisms for detecting early sickness, a shortage of doctors, nurses, laboratory diagnostics, and imaging facilities due to the burden of COVID-19 cases, the absence of systematic animal surveillance and coordination with veterinary services, and patients hesitant to visit hospitals due to fear of acquiring SARS-CoV-2 infection are some other challenges in the management of known and newly emerging zoonotic infections during the COVID-19 pandemic^51^.

Such diseases have forced medical organizations to prioritize their goals, focus on eradicating them, and develop better vaccination schedules and programs. Measures towards various diseases like tuberculosis, malaria, AIDS, etc., result in insufficient funds for research of zoonoses, thus cutting out on the preventive measures of zoonoses. Some developed countries follow a strict protocol, which benefits in eradicating zoonotic diseases. The population is highly unlikely to change their habits or beliefs, which causes the attempts to eliminate the disease to go awry. It also should not be forgotten that if another pandemic strikes, our world could suffer a reverse in human development^51^. While there is a raging race among many countries and pharmaceutical giants to make COVID-19 drugs, vaccines, diagnostic tests, and personal protective gear, zoonotic diseases are wholly neglected as they are not as contagious and affect mainly middle-to low-income nations and are not as lucrative as the coronavirus pandemic pharmacological market, which plays a significant role in hampering research and development of drugs, diagnostics methods, and vaccines^52^.

### Strategies adopted by India during the COVID-19 pandemic that can help in future zoonotic disease pandemic control

In 2020, the sudden increase in COVID-19 cases shocked the entire world. It is difficult to assign blame and improper to highlight the failings if there was inaccurate information regarding the early surge in COVID-19 cases from the source, a lack of proper information from the WHO, inadequate prevention measures, or strong border controls at the beginning of the first wave. There were knowledge, healthcare professionals, infrastructural gaps, and education gaps. What is remarkable is how India and other South Asian nations handled the crisis^53,54^. There was an increase in instances in India due to various factors, including poor disease management skills, fear of emerging viruses, poor direction, and inadequate public understanding of the importance of proper education, and a lack of basic healthcare infrastructure, such as personal protective equipment. That occurred while the first wave was beginning. People and the government stepped up to address the issue and improve things. In the West, it could appear to be commonplace, yet it marked the beginning of a new era in medical practice in developing countries. Medical students and recent graduates were taught to work in the hospital’s COVID wards, ICU, and COVID screening booths. With the development of specialised COVID hospitals and the transformation of public spaces into outpatient clinics. Additionally, they were stationed in COVID-19 camps outside of the hospitals. Doctors started YouTube channels to educate people about the virus, how to recognize symptoms, the value of social isolation and COVID-19 checks, medication, and its uses. Applying the recommendations from textbooks and academic texts to real-world situations is challenging when there is a heavy population burden. Government action was strictly enforced, resulting in several tight lockdowns that were gradually lifted, much like tapering steroid doses. Intense vaccination campaigns gave away free vaccines to high-risk individuals, medical professionals, and eventually, the general public. They also provided education, highlighted vaccine safety through YouTube channels and social media, and digitally linked vaccine doses to Indian identity cards (Aadhar cards). These lines, ‘Water water everywhere but not a drop to drink’, are well-known in poems. There was air-air everywhere during the second wave, but not enough oxygen for breathing. Cylinders of oxygen were scarce all over the nation. To give patients the required care, the Indian government and numerous public-private partnerships have developed new programs for rapidly establishing oxygen plants. There are internet resources like Swastham that provide details regarding the availability of hospital beds, medications, and doctors that provide comprehensive care. Leading medical professionals began providing online therapy to help educate and assist the public. The idea of digital health was changed by work-from-home and online modes. A small sector that produces equipment, sanitizer, and masks is growing. Some details on the realistic strategies adopted by India that can serve as a model for future pandemics and emergent conditions are mentioned as follows:

1. Strengthen the healthcare system: education and training of medical professionals to work in COVID screening camps, hospital wards, and the ICU; providing PPE; building oxygen plants; effective indigenous vaccines; digitalizing India that helped to provide treatment and counseling from grass root level; COVID-19 dedicated hospitals; improved access to services; robust measures to collaborate various specialties to provide care, increased coordination and information among healthcare facilities, laboratories, and public health for early response; strengthening research development through collaboration.
2. Public awareness and education with International cooperation: mass communication; preparedness, and resource planning; providing vaccines not only to the home country but increasing production to provide support to other countries.
3. Strict implementation of rules and regulations- COVID-19 lockdowns, border control, and travel restrictions.
4. Data sharing and digital technology: work-from-home; online care and support; linked COVID-19 dose to the Aadhar card; allowing entry to public spaces and travel after vaccines details are verified from the identity card.
5. Community engagement and empowerment: a public-private partnership, setting up oxygen plants, social media use, the rise of small industries, and supporting small setups.
6. Strengthening OH approach: India believes in the principle of ‘Vasudhaivakutumbakam’, which led to providing support to other needy countries in times of need.

**Figure 2:**
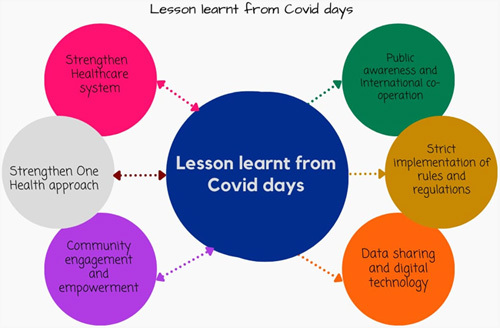
One Health - Coordination, communication, collaboration at the local, national, and global level to achieve the best outcomes for people, animals, plants, and the environment.

To achieve the best health outcomes for humans, animals, plants, and our environment, India’s OH approach (Fig. 2) premise calls for communication, coordination, and collaboration^48^. OH hub is in the developing stage. The development of a protocol for the database of zoonotic disease research in India, the national standing committee for zoonotic diseases, programs to control zoonotic and infectious diseases, the Make in India initiative, and other initiatives support this principle. Every coin has two sides, and there are still obstacles to achieve the goals, such as a lack of surveillance, adequate cooperation with agriculture and animals, improper implementation of the OH hub, and a failure to pay attention to wild zoonosis. To battle emerging illnesses and improve public health, it is vital to have a strong interdisciplinary focus and to be flexible, collaborative, and always learning.

### Recommendations and call of action

The stability of ecosystems has been significantly harmed by population explosion and anthropogenic activity, allowing viruses like COVID-19 to spread freely among humans^55^. Zoonotic illnesses are a severe threat to public health. Both natural and anthropogenic activities that release zoonoses can upset the delicate balance between the agent, host, and environment at any time. With our current understanding, we cannot precisely anticipate when or how severe the next zoonoses pandemic will be due to the inevitability of their emergence in the future. For our preparation to survive such a pandemic and to prevent the spread of similar pandemics in the future, the following preventive measures must be assured^3,6^:

1. Local and international regulatory agencies must design and execute efficient disease control procedures to lessen the danger of exposure to humans to wild animals^41^.
2. Monitoring and conserving animals and developing an effective systematic animal surveillance system.
3. With modern techniques like satellite-based remote sensing systems and molecular epidemiological tools, active and broader surveillance of zoonotic diseases and monitoring is possible. Incorporating modern information technology and creating mobile applications to provide online consultation to mild to moderate cases to limit COVID-19 spread and treat endemic zoonotic infections.
4. Ensure that animal-based food is produced safely and limit open meat markets as much as possible, especially in developing and underdeveloped countries.
5. Campaigning to raise public awareness of zoonoses by roping in mass media and the internet- government-funded announcements in public interest circulated via print and television media to educate the public about the dangers of zoonoses, especially during the COVID-19 pandemic^3^.
6. Policymakers in the tropics must make sure that their measures do not increase the risks posed by COVID-19 or any upcoming pandemics^56^.
7. Limit the spread of infections, economic losses, and eventually, human losses. Improved systems for animal health should be implemented, with tougher sanitary controls at the quarantine zones and borders^57^.
8. Involve prominent individuals and political figures involved in public health problems to educate people about zoonotic infections, quarantine, and prevention measures.
9. The government must provide exclusive research grants to encourage drug, diagnostic methods, and vaccine development targeting endemic zoonotic infections aiming for the total eradication of the disease.
10. Outreach programs to identify vulnerable populations (children, aged, immunocompromised, malnourished population) conducted in schools, colleges, stadiums, open marketplaces, and town halls should be conducted. Vaccination programs should be expedited for such vulnerable groups, and special protection should be provided^51^.

## Conclusion

Emerging zoonotic infection outbreaks are getting documented in various parts of the world and are a potential threat for causing the next global pandemic or major epidemics. The economic and health infrastructure of the affected countries is on the verge of collapse by these outbreaks, with high morbidity and mortality rates and national economic instability. Timely preparedness, creating awareness, and early intervention will help in containing the outbreaks. Studying and eliciting the factors that influence the outbreak and improving the understanding of zoonotic infection dynamics is essential. It is indispensable to devise appropriate preventive measures that could prevent future outbreaks.

## Ethical approval

Not applicable.

## Consent

Not applicable.

## Sources of funding

None.

## Author contribution

MB: conceptualization, project administration, supervision writing –original draft, writing –review and editing; I.R., S.R., S.P., P.B., S.A., S.S., A.S., S.M., and A.A.: conceptualization, project administration, writing – original draft, writing – review and editing; P.R.: project administration, writing – review and editing. All authors are accountable for all the aspects of this work.

## Conflicts of interest disclosure

The authors have no conflicts of interest to declare.

## Research registration unique identifying number (UIN)

1. Name of the registry: not applicable.
2. Unique Identifying number or registration ID: not applicable.
3. Hyperlink to your specific registration (must be publicly accessible and will be checked): not applicable.

## Guarantor

Mainak Bardhan.

## Data availability statement

Not applicable.

## Provenance and peer review

Not commissioned, externally peer-reviewed.

## Acknowledgements

None.
